# Monitoring Macro- and Microcirculation in the Critically Ill: A Narrative Review

**DOI:** 10.1055/s-0043-1772175

**Published:** 2023-09-05

**Authors:** Syed Nabeel Muzaffar, Akshyaya Pradhan, Suhail Sarwar Siddiqui, Shubhajeet Roy, Timil Suresh

**Affiliations:** 1 Department of Critical Care Medicine, King George's Medical University (KGMU), Lucknow, Uttar Pradesh, India; 2 Department of Cardiology, King George's Medical University (KGMU), Lucknow, Uttar Pradesh, India; 3 Faculty of Medical Sciences, King George's Medical University (KGMU), Lucknow, Uttar Pradesh, India

**Keywords:** circulatory shock, hemodynamic monitoring, fluid responsiveness, personalized hemodynamic monitoring

## Abstract

Circulatory shock is a common and important diagnosis in the critical care environment. Hemodynamic monitoring is quintessential in the management of shock. The currently used hemodynamic monitoring devices not only measure cardiac output but also provide data related to the prediction of fluid responsiveness, extravascular lung water, and also pulmonary vascular permeability. Additionally, these devices are minimally invasive and associated with fewer complications. The area of hemodynamic monitoring is progressively evolving with a trend toward the use of minimally invasive devices in this area. The critical care physician should be well-versed with current hemodynamic monitoring limitations and stay updated with the upcoming advances in this field so that optimal therapy can be delivered to patients in circulatory shock.

## Introduction

Hemodynamic monitoring is an essential tool to understand the etiology and pathophysiology underlying various shock states, and thereby, administer life support therapies (fluid resuscitation, vasopressors, or inotropes) in an appropriate and timely manner. 1 2 3 Hemodynamic monitoring devices is broadly divided into those pertaining to macrocirculation and microcirculation.

## Assessment of Macrocirculation

**Fig. 1:**
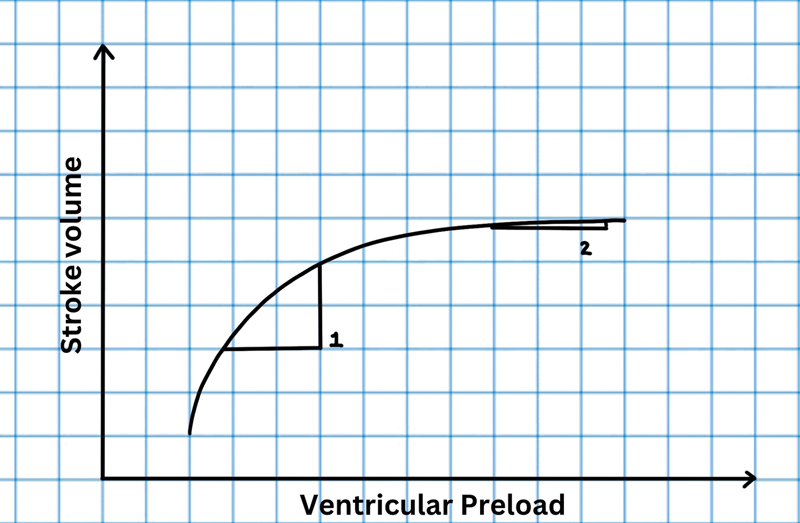
Frank-Starling curve. Patient at point 1 (steep part of curve) is more fluid responsive than patient at point B (flat part of curve).

Fluid resuscitation is usually the first step in the management of circulatory shock. Fluid responsiveness has been defined as > 15% increase in stroke volume (SV) within 15 minutes of 250 to 500 mL or 6 mL/kg crystalloid over 20 to 30 minutes. 1 2 Traditionally, decisions for fluid responsiveness have relied upon assessment of clinical parameters (peripheral pulse, heart rate, noninvasive blood pressure (BP), urine output, and fluid balance), central venous pressure (CVP), 4 5 and pulmonary artery occlusion pressure (PAOP) 6 7 8 monitoring. But there are numerous fallacies of these methods. Extremes of CVP (too low or too high) will miss correct fluid responsiveness or lack of it in many cases. A large meta-analysis conducted in 2013, which included 22 studies from intensive care unit (ICU) and 22 from operating room, did not support the use of CVP for guiding fluid administration. 9 The use of pulmonary artery catheter (PAC) has also decreased since its introduction in the 1970s after randomized trials failed to show an improvement in outcome of patients with PAC. 10 11 12 Moreover, volumetric indices for end-diastolic volumes (like right ventricular end-diastolic volume, global end-diastolic volume [GEDV], and left ventricular end-diastolic area index) may be influenced by diastolic compliance. 13 Some of the reasons why these pressometric (CVP/delta CVP/PAOP) and volumetric indices of hemodynamic monitoring, also known as static indices of preload, 14 poorly predict fluid responsiveness are 1 2 3 4 15 :

- Frank-Starling principle: As per the Frank-Starling principle, the heart adjusts its SV on the basis of its sarcomere length (end-diastolic volume or preload). This relationship between SV and preload is curvilinear. As preload increases, SV increases until a point beyond which SV shows no further change to increasing preload. So, a favorable hemodynamic response to fluids can be expected only if the heart is operating in the steep part of the curve ( Fig. 1 ).
- Fluid challenge 16 17 18 may be a good way to find out if a patient is fluid responsive or not (position on steep or flat part of the Frank-Starling curve), although only half of the critically ill population may be fluid responsive. The initial fluid challenge technique was described by Weil and Henning in 1979 which comprised of 2 to 5 rule for CVP and 3 to 7 rule for PAOP. 19 Later on, a modified fluid challenge technique was proposed by Vincent and Weil in 2006 including four components: type of fluid, rate of infusion, desired therapeutic response, and assessment of safety limits. 20 Fluid challenge might be used in clinical scenarios unless for obvious safety concerns such as florid heart failure, refractory hypoxemia, massive fluid overload, etc.

## Dynamic Indices of Preload to Predict Fluid Responsiveness

In the 2000s, Marik et al 21 introduced the concept of dynamic indices of fluid responsiveness, associating respiratory variations in arterial BP with volume status of patient and hence, the effects of volume expansion on cardiac index. Later on, Cavallaro et al 22 classified these indices into three groups as described in Table 1 . All these indices are known as dynamic indices of preload. 21 22 23 24 A brief description of some of these indices has been mentioned below.

**Table 1:** Dynamic indices of preload

Dynamic indices of preload		
Group A MV-induced variation in SV or SV-derived parameters	Group B MV-induced variation in nonstroke volume-derived parameters	Group C Preload redistributing maneuvers different from standard MV
• Systolic pressure variation (SPV) • Stroke volume variation (SVV) • Pulse pressure variation (PPV) • Ventilation induced- plethysmography variation (VPV) • Peak aortic flow velocity (delta Vpeak) • Aortic blood flow variation (delta ABF)	• Respiratory variability of inferior and superior vena cava • Left ventricular pre-ejection period variation (delta PEP)	• PLR-induced change in cardiac output (PLR-cCO) • Respiratory systolic variation test (RSVT) • End expiratory occlusion test (EEOT) • Valsalva maneuver

Abbreviations: ABF, aortic blood flow; EEOT, end-expiratory occlusion test; MV, mechanical ventilation; PEP, pre-ejection period; PLR, passive leg raising; PLR-cCO, PLR-induced change in cardiac output; PPV, pulse pressure variation; RSVT, respiratory systolic variation test; SPV, systolic pressure variation; SV, stroke volume; SVV, stroke volume variation, Vpeak, peak aortic flow velocity; VPV, ventilation-induced plethysmographic variation.

### Physiology Behind Group A and B Indices 23

To understand the physiologic rationale behind group A and B indices, it is important to recapitulate the concept of heart-lung interaction within a closed thoracic cavity. During inspiratory phase of controlled positive pressure ventilation, there is increased intrathoracic pressure (ITP) causing decrease in preload to right ventricle (RV) and as a result of decreased venous return. Thus, the blood volume being ejected in that cardiac cycle from RV decreases, which leads to decreased PA blood flow, left ventricle (LV) filling, and hence, fall in LV SV, which manifests in expiration due to a delay caused by pulmonary circuit transit time. Simultaneously, in the same inspiratory cycle LV SV increases due to decreased LV afterload and squeezing of the alveolar vessels into the left atrium. Therefore, phasic variations are noticed in positive pressure ventilation, which gets exaggerated in hypovolemia. Larger changes are noticed when patient is lying on steep portion of the Frank-Starling curve.

## Systolic Pressure Variation

**Fig. 2:**
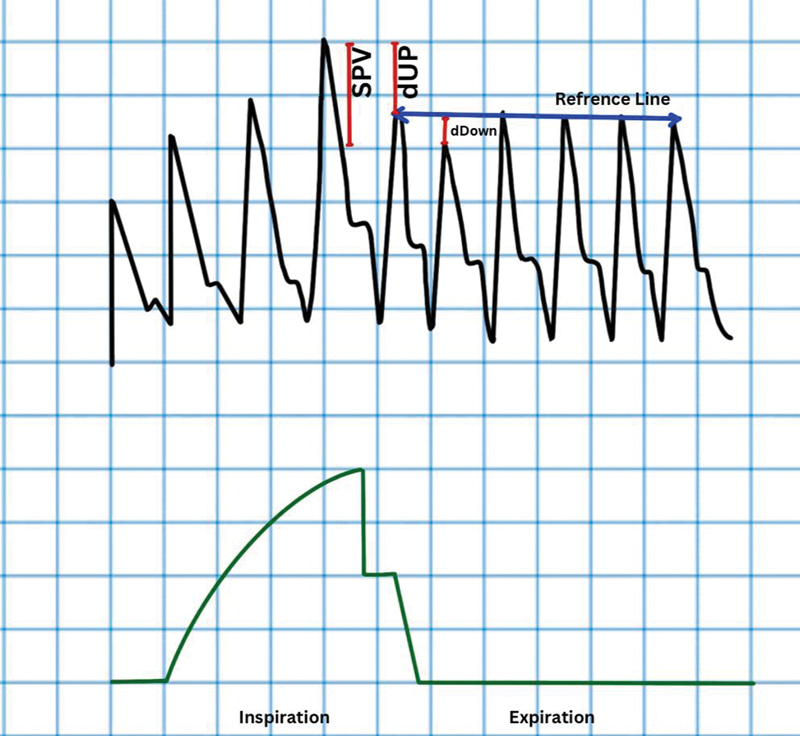
Systolic pressure variation (SPV). dDown, delta down; dUP, delta UP.

Systolic pressure variation (SPV) is calculated directly from arterial pressure waveform as SPV = maximum systolic pressure (SPmax) – minimum systolic pressure (SPmin) or as a percentage, SPV% = [(SPmax – SPmin)/ ½ (SPmax + SPmin)] × 100 ( Fig. 2 ). SPV can be further divided into two components, delta up (dUp) and delta down (dDown), using a reference systolic pressure (SPref) measured during end-expiratory pause so that dUp = SPmax – SP ref and dDown = SPref – SPmin. dDown a more reliable indicator of fluid responsiveness because it reflects the expiratory decrease in LV SV related to inspiratory decrease in RV ejection fraction. A cutoff of 8.5 mm Hg has a sensitivity of 82%, specificity of 86%, and area under the receiver operating curve (AUC) of 0.92 for predicting fluid responsiveness. dDown threshold of 5 mm Hg has AUC of around 0.97. 23 25

## Stroke Volume Variation

**Fig. 3:**
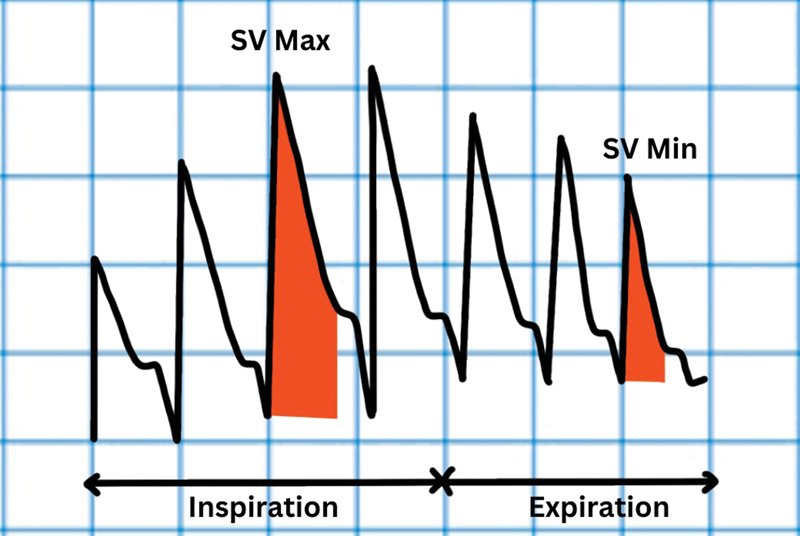
Stroke volume variation (SVV). SVmax, maximum stroke volume; SVmin, minimum stroke volume.

Stroke volume variation (SVV) calculates the difference between SV during the inspiratory and expiratory phases of respiration ( Fig. 3 ). It was earlier calculated using aortic probes but now-a-days, SVV can be directly estimated by pulse contour analysis based cardiac output (CO) monitoring devices (like PiCCO, LiDCO, and FloTrac). SVV% = [(SVmax – SVmin)/ ½ (SVmax + SVmin)] × 100. SVV threshold of 9.5 to 11.5% has AUC of 0.87 to 0.88 for predicting fluid responsiveness. 23 26

## Pulse Pressure Variation

**Fig. 4:**
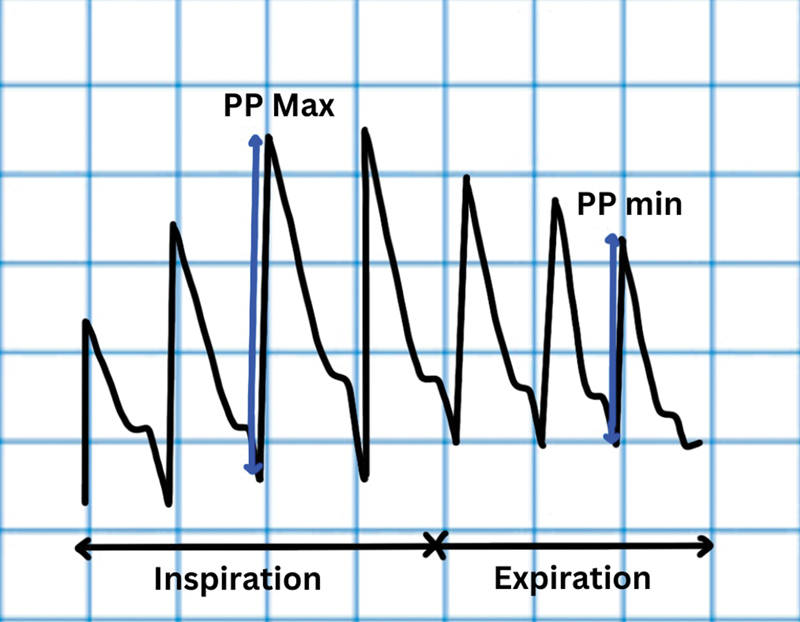
Pulse pressure variation (PPV). PPmax, maximum pulse pressure; PPmin, minimum pulse pressure.

Pulse pressure is the difference between arterial systolic and diastolic pressures. It is influenced by SV and aortic compliance. As comparison of pulse pressures is done over a single respiratory cycle, change in arterial compliance is considered to be minimal. Pulse pressure variation (PPV) can be calculated either directly from the arterial waveform or it can be directly recorded by PiCCO ( Fig. 4 ). PPV% = [(PPmax – PPmin)/ ½ (PPmax + PPmin)] × 100. PPV threshold of 13% has AUC of 0.98 with 94% sensitivity and 96% specificity for predicting fluid responsiveness. 23 In a systematic review of 29 studies by Marik et al in 2009 on the role of dynamic changes in arterial waveform variables in predicting fluid responsiveness, PPV was found to have better AUC, sensitivity, specificity, and likelihood ratio as compared to SPV, SVV, and some static parameters of preload. 21

Two large multicenter studies (around 800 patients, 62 ICUs) have recently evaluated the utility of PPV and observed that PPV is suitable in 1 to 2% ICU patients only. 27 28 In these studies, among those on controlled ventilation one of the important inhibiting factors to institution of PPV was use of lung protective ventilation (tidal volume < 8 mL/kg ideal body weight [IBW]). Recently, Myatra et al have devised a test called “Tidal Volume Challenge” to assess fluid responsiveness in settings of low tidal volume mechanical ventilation (MV), in which the tidal volume is transiently increased to 6 to 8 mL/kg IBW for 1 minute to assess SVV/PPV with a cutoff of 2.5 and 3.5% taken as thresholds for fluid responsiveness for SVV and PPV, respectively. 29

## Respiratory Variation in Inferior Vena Cava/Superior Vena Cava

Inferior vena cava (IVC) and superior vena cava (SVC) are distensible blood vessels. Their diameter and flow vary with respiration and the changes in size are exaggerated by hypovolemia. 30 31 32 IVC enters the right atrium (RA) immediately after crossing the diaphragm so its intramural pressure is similar to RA pressure while its extramural pressure represents intra-abdominal pressure (IAP). In positive pressure ventilation, pleural pressure gets transmitted fully to RA and partially to the abdomen. Therefore, IVC gets distended during inspiratory phase of positive pressure ventilation. IVC distensibility can be measured by transthoracic echocardiography or transabdominal ultrasonography. IVC distensibility index is calculated as dIVC = [(Dmax – Dmin)/(Dmin)] × 100. dIVC > 18% has been shown to have AUC of 0.91 for predicting fluid responsiveness. 30 31

Unlike IVC, SVC has mainly an intrathoracic course. Positive pressure ventilation decreases its transmural pressure, and hence, SVC gets collapsed. SVC collapsibility index can be calculated using transesophageal echocardiography or esophageal Doppler and as cSVC = [(Dmax – Dmin)/(Dmax)] × 100. cSVC > 36% has been found to have AUC of 0.99 for predicting fluid responsiveness. 30

However, there are some limitations of group A and B indices which restrict their applicability in critically ill patients, as mentioned below 23 :

1. Positive pressure, controlled ventilation, sinus rhythm, and large tidal volume ≥ 8 mL/kg are needed to ensure sufficient change in ITP for correct assessment of these indices.
2. Further studies are needed to validate these indices in the setting of vasoactive drugs and in open chest or abdomen conditions.
3. In scenarios like cardiac tamponade, RV failure, raised IAP, and arrhythmias.
4. Morbid obesity and postlaparotomy conditions, assessment of IVC/SVC changes is difficult.

## Group C Indices

### Passive Leg Raising 33 34 35

Lifting legs in circulatory collapse has been used by first aid rescuers for many years. In passive leg raising (PLR), there is a gravitational transfer of around 300 mL blood from the lower part of the body toward the central circulatory compartment which acts as a fluid challenge. Moreover, any change in CO vanishes completely once the legs are returned back to horizontal position. Thus, PLR acts as a “self and reversible” volume challenge. PLR-induced change in aortic blood flow of 8 to 10% has been found to have AUC of 0.91 to 0.96 for predicting fluid responsiveness. Studies have shown that PLR-induced change in CO (PLR-cCO) is at least as accurate as PPV and is better than SVV and SPV for predicting fluid responsiveness. 35 Another advantage of PLR is that it is reliable in conditions where other indices of fluid responsiveness fail, like spontaneous respiration, arrhythmias, low tidal volume ventilation, and low lung compliance. 32 36 37

There are certain essential points that need to be kept in mind while doing PLR:

1. PLR should start from semirecumbent position (and not from supine position).
2. PLR effects must be assessed by direct measurement of CO (and not by simple measurement of BP).
3. Technique used to measure CO must be able to detect the short and transient changes induced by PLR as effects of PLR may disappear after 1 minute.
4. CO should be measured not only before and during PLR but also when patient is moved back to semirecumbent position to check return of CO to baseline.
5. Avoid pain, cough, discomfort, and awakening-induced adrenergic stimulation (adjust bed and do not raise patient's legs, explain procedure to conscious patients, and aspirate tracheal secretions).

**Fig. 5:**
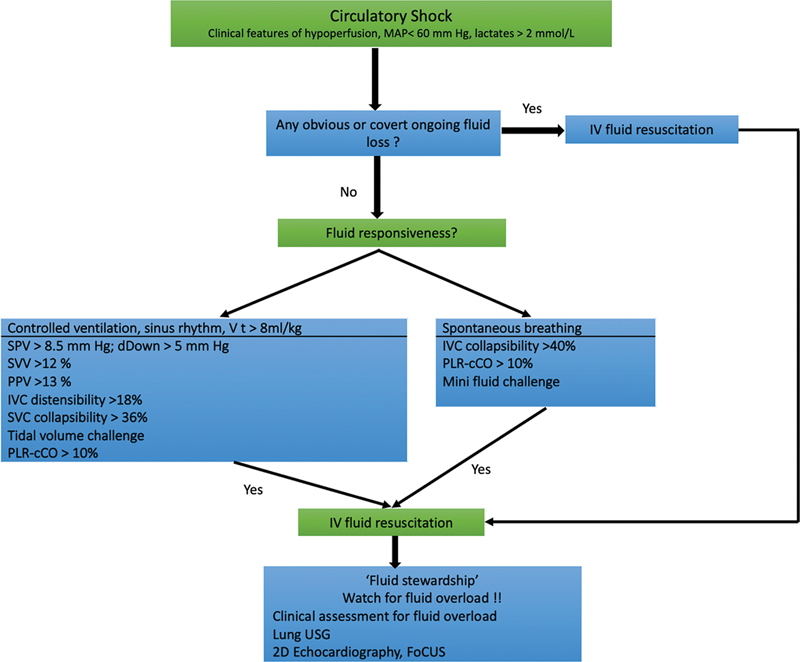
Algorithm for guiding fluid resuscitation in patients with shock on the basis of fluid responsiveness. SPV, systolic pressure variation; SVV, stroke volume variation; PPV, pulse pressure variation; PLR-cCO, passive leg raising-induced change in cardiac output; FoCUS, focus cardiac ultrasound; Lung USG, lung ultrasonography.

An algorithmic approach to assessment of fluid responsiveness has been shown in Fig. 5 .

### Cardiac Output Monitoring 38

Adolf Fick described about CO estimation in 1870 for the first time. Until the introduction of PAC, Fick's principle was the reference standard for CO determination. Since the last decade, many noninvasive CO monitors have been introduced which do not require the introduction of PAC, as shown in the classification of CO monitoring devices in Table 2 .

**Table 2:** Classification of cardiac output monitoring devices

Classification of cardiac output (CO) monitoring devices
Invasive CO monitoring
• PAC: intermittent bolus thermodilution (gold standard)
Minimally invasive CO monitoring
• Esophageal Doppler • Pulse contour analysis • Calibrated: Transpulmonary thermodilution: PiCCO (Pulsion Medical Systems, Munich, Germany), LiDCO (LiDCO Group Plc, London, U.K.) • Noncalibrated: FloTrac (Vigileo, Edwards Life Sciences, Irvine, California, United States)
Noninvasive CO monitoring
• Thoracic bioimpedance • Partial CO _2_ rebreathing system (NICO, Respironics, Murrysville, Pennsylvania, United States) • Pulse wave analysis (noninvasive): ClearSight system, CNAP system • Pulse wave transit time (PWTT): Nihon Kohden

Abbreviations: CO, cardiac output; LiDCO, lithium dilution cardiac output; NICO, noninvasive cardiac output monitor; PAC, pulmonary artery catheter; PiCCO, pulse contour cardiac output.

## Invasive Cardiac Output Monitoring

### Pulmonary Artery Catheter 7

PAC is still the reference gold standard method which calculates CO using intermittent (or semicontinuous) bolus thermodilution (TD) method. Five to 10 mL cold saline (≤ 25°C) is injected into the RA which mixes with venous blood and cools down. Change in temperature is recorded in PA by a thermistor near the tip of the PAC. Thus, a TD curve is generated which calculates CO by Stewart-Hamilton equation. An average of three values is finally taken as CO. PAC also provides data regarding right atrial pressure, PA pressures, PAOP, and mixed venous oxygen saturation (SvO2). It has certain limitations, including invasiveness, possibility of complications while insertion, difficulty in data interpretation, and lack of evidence in favor of PAC. PAC still has limited use in the management and diagnosis of pulmonary hypertension, undifferentiated shock, cardiogenic shock, heart failure, and obscure hemodynamics (e.g., congenital heart disease).

## Minimally Invasive Cardiac Output Monitoring 39

### Esophageal Doppler 40 41

Esophageal Doppler monitors CO by measuring blood flow in the descending thoracic aorta. In sedated and MV patients, esophageal Doppler probe is introduced either by oral or by nasal route and fixed at 35 to 40 cm from teeth. The tip of the probe is then rotated to face the descending aorta. The characteristic velocity signal from the descending aorta appears as a swoosh sound. D-shaped piezoelectric crystal is present at the tip of the probe which acts as Doppler transducer and transmits sound waves either as 4 MHz continuous wave or as 5 MHz pulsed wave. Signals reflected back by red blood cells (RBCs) are then recorded to calculate Doppler shift.

Estimation of aortic blood flow is done by measurement of velocity time integral (VTI). SV is then calculated by the formula, SV = VTI × cross-sectional area (cross-sectional area is determined from the aortic diameter which in turn is calculated by a nomogram based on age, weight, and height [Cardio Q] or by M mode echo [HemoSonic 100]).

There are certain assumptions in esophageal Doppler monitoring system which limits its applicability:

1. Proportion of blood flow in ascending and descending aorta is assumed to be fixed and “flat” (same RBC speed).
2. Aortic diameter is considered to be fixed during systole.
3. Angle between ultrasound beam and blood flow is assumed to be fixed (45–60 degrees).
4. A fixed proportion of 30% blood flow is thought to be diverted to the heart, brain, and limbs before reaching the descending thoracic aorta.

## Transpulmonary Thermodilution and Pulse Contour Analysis 42

Two different proprietary devices use transpulmonary TD (TPTD) techniques, namely: PiCCO (Pulsion Medical systems, Germany) and VolumeView (Edwards Lifesciences, USA). These devices come in the category of minimally invasive devices as they are less invasive than PAC, which traverses through the pulmonary vasculature and calculates intermittent CO using thermistor lodged in the PA.

TPTD is done for external calibration of system. Both central venous (internal jugular vein /subclavian vein) cannulation and femoral/axillary artery cannulation are required. A known volume of ice cold saline injection is injected into the central vein and changes in blood temperature are recorded in femoral or axillary artery. TD curve is generated and CO is estimated from the curve using Stewart-Hamilton equation similar to PAC (average of three values is recorded). Other important variables recorded are GEDV (preload), cardiac function index (contractility), extravascular lung water (EVLW), SVV, PPV and pulmonary vascular permeability index.

After calibration by TPTD, pulse contour analysis is done which is based on the Windkessel model. This model states that the volume of blood entering a vessel of infinite length is equal to the volume of blood leaving that vessel over the period of cardiac contraction. During systole, vessels expand while during diastole, they contract. The aorta acts as a capacitor and systemic arterioles act as resistors.

Based on the relationship among BP, SV, arterial compliance, and systemic vascular resistance, SV is calculated as the area under systolic portion of arterial waveform as an integral of change in pressure (P) from end-diastole (t0) to end-systole (t1) over time and is inversely proportional to impedance ( *Z* ) of the aorta.

Arterial compliance is calculated from the shape of diastolic portion of arterial pressure waveform.

TPTD devices have good agreement with PAC but require frequent calibration and suffer from the limitations inherent in any monitoring system based on arterial pressure waveform analysis.

These devices give continuous and real-time CO monitoring and other fluid responsiveness data and are calibrated devices. They use two methods of CO measurement, namely, TPTD and arterial pulse contour analysis. While arterial pressure contour analysis gives continuous and real-time CO, TPTD is used to externally calibrate and thus it is more reliable in critical care settings as compared to uncalibrated CO monitors.

### LiDCO 43 44

LiDCO is another pulse contour analysis method based on lithium indicator dilution in which LiCl 0.002 to 0.004 mmol/kg is injected via central or peripheral vein. There is a lithium sensor attached to the peripheral arterial line. Three milliliters of blood is withdrawn through the arterial line and a lithium time (dye dissipation) curve is generated from which CO is analyzed by Stewart-Hamilton equation. This method is as reliable as other TD methods but is unable to predict CO accurately in patients on neuromuscular blockers and those taking therapeutic lithium, and also, there are chances of anemia due to frequent blood sampling and overall lithium is costly. 27

### FloTrac System 45 46

FloTrac system is also a pulse contour analysis method which includes FloTrac sensor and Vigileo monitor and requires only a peripheral arterial catheter (usually radial artery catheter) for CO estimation. It does not require any external calibration. A proprietary algorithm analyzes the arterial pressure waveform and samples it at 100 Hz and updates it every 20 seconds. Characteristics of the arterial waveform are then coupled with patient's demographics to calculate CO. This algorithm operates on the principle that pulse pressure is proportional to SV and inversely proportional to vascular compliance. Continuous self-calibration goes on by an automatic vascular tone adjustment property of software algorithm which eliminates the need for any external calibration.

But FloTrac does not track changes in SV accurately and has poor agreement with PAC. This device is a noncalibrated device and may not be very suitable for prolonged use in critical care environment than for short-term use in operating rooms. 28

## Noninvasive Cardiac Output Monitoring

### Partial CO _2_ Rebreathing System 47 48

Partial CO _2_ rebreathing system is used which consists of CO _2_ infrared sensor, airflow/pressure pneumotachometer, pulse oximeter, and a disposable partial rebreathing loop. According to the Fick principle, CO is calculated by the following formula:

CO = Change in VCO _2_ /Change in CaCO _2_

where VCO _2_ = CO _2_ clearance, and CaCO _2_ = arterial CO _2_ content (estimated from end-tidal CO2).

However, this system measures only pulmonary capillary blood flow and is inaccurate in high shunt conditions. It is not applicable in low minute ventilation conditions and for spontaneously breathing patients.

### Thoracic Bioimpedance 49

A high-frequency, low-magnitude electric current of known frequency and amplitude is applied across thorax and changes in voltage are measured. The ratio between voltage and current amplitude is then calculated to determine impedance (Zo). SV is proportional to product of maximal rate of change of Zo and ventricular ejection time (VET) (VET is calculated from electrocardiography). Electrical resistance of thorax is assumed to be directly related to intrathoracic blood volume.

It is inaccurate in ICU settings (excess body motion, noise, and excess EVLW). Moreover, Zo depends on electrode placement, body size, temperature, and humidity.

Pulse wave analysis by finger-cuff (volume clamp technique; ClearSight and CNAP systems) and pulse wave velocity (based on pulse wave transit time) are other noninvasive techniques to monitor CO but they have not been validated for clinical usage. 48 49

**Fig. 6:**
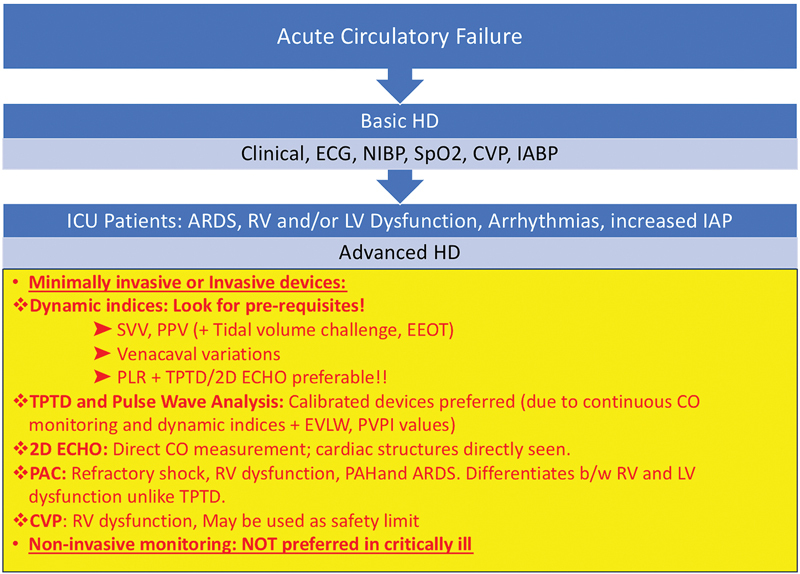
Hemodynamic monitoring devices in intensive care unit (ICU) settings. ECG, electrocardiography; NIBP, noninvasive blood pressure; SpO2, oxygen saturation; CVP, central venous pressure; IABP, invasive arterial blood pressure; SVV, stroke volume variation; EEOT, end-expiratory occlusion test; PLR, passive leg raising; TPTD, transpulmonary thermodilution; EVLW, extravascular lung water; PVPI, pulmonary vascular permeability index; CO, cardiac output; RV, right ventricle; PAH, pulmonary artery hypertension; LV, left ventricle; ARDS, acute respiratory distress syndrome; PAC, pulmonary artery catheter.

Table 3 shows the merits and demerits of various hemodynamic monitoring devices and Fig. 6 shows which hemodynamic monitoring device may be preferable in various ICU settings.

**Table 3:** Merits and demerits of various hemodynamic monitoring devices

Tool	Accuracy	Other variables besides CO	Mean % error (vs. TD)	Demerits
• **PAC: Gold standard; calibrated; directly measures right heart and PA; but invasive**				
• **Minimally invasive devices**				
	**Reliability**	**Additional features**	**% Error**	**Demerits**
TPTD (calibrated) e.g., PiCCO	Good	ITBV, GEDV, CFI, PVPI, EVLW	Accurate	Damping, aortic diameter variability, aortic valve and vessel pathology, cannot differentiate between RV and LV dysfunction
LiDCO (calibrated)	Good	−	41	Li, NMBs Frequent sampling
PRAM (noncalibrated)	Fair	−	41	For stable patients only
Esophageal Doppler	Fair	Yes; preload Contractility, afterload; SVV		Aortic size assumptions Probe position, only descending aortic flow; sedated and MV patients only
• **Noninvasive devices**				
ClearSight	Less	−	44	Noncalibrated
PWTT	Less	−	62	Noncalibrated
CO _2_ rebreathing	Less	−	40	Noncalibrated, healthy lungs only, for sedated patients on controlled MV only
Bioimpedance	Less	−	40–45	Noncalibrated, for stable patients only

Abbreviations: CFI, cardiac function index; CO, cardiac output; CO _2_ rebreathing, carbon dioxide rebreathing; EVLW, extravascular lung water; GEDV, global end-diastolic volume; ITBV, intrathoracic blood volume; LiDCO, lithium dilution cardiac output; LV, left ventricle; MV, mechanical ventilation; NMBs, Na-metal batteries; PA, pulmonary artery; PAC, pulmonary artery catheter; PiCCO, pulse contour cardiac output; PRAM, pressure recording analytical method; PVPI, pulmonary vascular permeability index; PWTT, pulse wave transit time; RV, right ventricle; SVV, stroke volume variation; TPTD, transpulmonary thermodilution.

## Echocardiography

Echocardiography is an important, noninvasive, repeatable, bedside tool to examine and monitor cardiac structure and function 50 51 52 and provides comprehensive information about the following parameters:

1. Preload estimation (end-diastolic volumes)
2. Fluid responsiveness indices: IVC/SVC assessment, left ventricular outflow tract VTI variation, and ascending aortic blood velocity/flow variation by pulsed wave Doppler
3. Systolic and diastolic function of LV and RV
4. Others: Regional wall motion abnormality, valves, thrombus, and pericardial effusion

Two-dimensional echocardiography along with critical care ultrasonography is increasingly gaining importance in emergency and ICU settings for timely diagnosis and management of shock as shown by Kanji et al in 2014 53 and Atkinson et al in 2018 54 .

Table 4 shows the application of abovementioned hemodynamic monitoring devices in different pathophysiological conditions.

**Table 4:** Application of hemodynamic monitoring devices in different pathophysiological conditions

	SPV, SVV, PPV	Vt challenge	IVC SVC	PLR-cCO	TPTD-EVLW	2D ECHO
Tidal volume (Vt) > 8 mL/kg IBW and controlled ventilation	Yes	−	Yes	Yes	Yes	Yes
Arrhythmias	−	−	Yes	Yes	Yes	Yes
ARDS	−	SVV/PPV + Vt challenge	Yes	Yes	Yes	Yes
Spontaneous ventilation	−	−	−	Yes	−	Yes (with PLR)
Open chest, e.g., intercostal chest drain (ICTD)	−	−	−	Yes	Yes	Yes
RV failure	−	−	−	Yes	Yes	Yes
Raised IAP	−	−	−	Yes	−	Yes

Abbreviations: ARDS, acute respiratory distress syndrome; IAP, intra-abdominal pressure; ICTD, intercostal chest drain; IVC, inferior vena cava; PLR, passive leg raising; PLR-cCO, passive leg raising-induced change in cardiac output; PPV, pulse pressure variation; RV, right ventricle; SPV, systolic pressure variation; SVC, superior vena cava; SVV, stroke volume variation; TPTD-EVLW, transpulmonary thermodilution-extravascular lung water; 2D ECHO, two-dimensional echocardiography.

## Tissue Oxygenation

Normal CO is frequently mistaken to represent normal flow of oxygen to vital organs. However, both are not the same. To assess the balance between oxygen delivery and metabolic demand of tissues, indicators of tissue oxygenation like venous oximetry, blood lactates, and capillary refill time (CRT) are commonly used.

## Lactates

Lactates are produced as an end product of glycolysis and are normally cleared out from the body by liver and kidneys. Hyperlactatemia is defined as serum lactate > 2 mmol/L and lactic acidosis is hyperlactatemia associated with metabolic acidosis (pH < 7.35). 55 High lactate levels generally indicate an underlying anaerobic metabolic state (secondary to poor oxygen delivery to tissues) leading to overproduction of lactates and are helpful in risk stratification and prognostication of patients. 56 57 58 Decrease in lactates (lactate clearance) with initial management strategies has been found to improve patient outcomes 59 60 and persistent hyperlactatemia should alarm the clinician to immediately address the adequacy of the underlying source of control measures. 55

There may be various causes of hyperlactatemia besides tissue hypoperfusion. Therefore, it is essential to approach and manage hyperlactatemia in the appropriate clinical scenario. 61

## Venous Oximetry

Central venous oxygen saturation (ScVO _2_ ) measured from central venous catheter with its tip at the junction of SVC and RA or mixed venous oxygen saturation (SvO _2_ ) (measured by distal tip of PA catheter) assesses the balance between oxygen delivery and consumption. 62 63 64 Changes in ScVO _2_ closely parallel that of SvO _2_ in critically ill patients. 65 ScVO _2_ < 70% indicates poor oxygen delivery to tissues. In 2001, the early goal-directed trial (EGDT) by Rivers et al showed a mortality benefit if ScVO _2_ > 70% was targeted as resuscitation endpoint. 66 However, the recent studies (ARISE, ProCESS, and ProMISe) 67 68 69 assessing the protocolized (goal-directed) resuscitation strategies targeting ScvO _2_ > 70% failed to show a mortality benefit pointing more toward the benefit of an individualized or personalized resuscitation strategy (focusing on early antibiotics and judicious fluid resuscitation) rather than focusing on a predefined and fixed target.

Besides lactates and venous oximetry, CO _2_ gap can provide further information by assessing the adequacy of regional blood flow. 70 71 In areas of decreased perfusion, CO _2_ diffuses through the tissues and accumulates in venous blood leading to increased venous CO _2_ content as compared to arterial CO _2_ content.

An example of CO _2_ gap is the PCO _2_ gap which measures difference in partial pressure of CO _2_ between central venous blood (P _v_ CO2) and arterial blood (P _a_ CO _2_ ). PCO _2_ gap (P _v_ CO _2_ –P _a_ CO _2_ ) > 6 mm Hg reflects poor regional blood flow.

## Peripheral Skin Perfusion

The compensatory sympathetic activation in shock diverts blood flow away from skin to vital structures like brain and heart. Moreover, there is no autoregulation in blood vessels of skin. So, skin perfusion assessment in the form of skin mottling (around the knee) 72 73 and CRT may play an important role in the assessment of tissue perfusion and oxygenation.

The recently conducted ANDROMEDA-SHOCK trial 74 compared peripheral perfusion-targeted strategy (by CRT at ventral part of the right index finger) to lactate-targeted resuscitation in early septic shock and found no significant difference between the two strategies in terms of 28-day mortality. However, the study was underpowered and unblinded and a Bayesian analysis of the ANDROMEDA study found mortality benefit with CRT-targeted resuscitation. 75 CRT may thus be considered as an alternative to lactate-targeted resuscitation pending further trials.

## Assessment of Microcirculation

Circulatory shock often involves disturbances of microcirculation, which comprises of vessels < 100 μm in diameter including arterioles, capillaries, and venules. Derangements of microcirculation include heterogeneity of blood flow (stagnant, intermittent on/off, obstructed or increased blood flow), reduced perfused vascular density, and impaired oxygen diffusion. 76 “Hemodynamic coherence” between microcirculation and macrocirculation is essential to ensure adequate tissue perfusion and oxygenation. 77

Hemodynamic indices described above assess the macrocirculation (global hemodynamic indices) and failed to assess microcirculation. Some of the important tools for assessing microcirculatory markers of perfusion include laser Doppler to measure RBC velocity in small tissues, near-infrared spectroscopy, orthogonal polarization spectral, and side-stream dark field imaging and assessment of sublingual microcirculation (hand-held video-microscopy). However, these tools are still under research. 78 79 80

## Conclusion

Hemodynamic monitoring is an integral part of critical care. Its major role is to optimize administration of fluids and vasoactive drugs at the right time in the right dose titrated as per personal needs of the patient. Since the last couple of years, our awareness about fluid responsiveness is improving. Many advanced CO monitors have turned up for optimally evaluating a patient's hemodynamics and the field is continuously evolving. As such, there is no monitoring device which is ideal for all patients and a good physician should embrace a combination of bedside clinical examination and corroborate it with data obtained from all these hemodynamic monitoring methods for judicious management of critically ill patients.
